# Gene or environment? Species-specific control of stomatal density and length

**DOI:** 10.1002/ece3.233

**Published:** 2012-05

**Authors:** Lirong Zhang, Haishan Niu, Shiping Wang, Xiaoxue Zhu, Caiyun Luo, Yingnian Li, Xinquan Zhao

**Affiliations:** 1 Department of Resource and Environment, Graduate University of Chinese Academy of SciencesBeijing 100049, China; 2 Institute of Tibetan Plateau Research, Chinese Academy of SciencesBeijing 100101, China; 3 Key Laboratory of Adaptation and Evolution of Plateau Biota, Institute of Northwest Plateau Biology, Chinese Academy of SciencesXining 810008, Qinghai, China

**Keywords:** Environmental control, genetic control, reciprocal transplant experiment, stomatal density, stomatal initiation, stomatal length

## Abstract

Stomatal characteristics are used as proxies of paleo-environment. Only a few model species have been used to study the mechanisms of genetic and environmental effects on stomatal initiation. Variation among species has not been quantified. In this paper, results from an in situ reciprocal transplant experiment along an elevation gradient in the northeast Tibetan Plateau are reported, in which the relative effects of genetics (original altitude) and environment (transplant altitude) on stomatal density (SD) and length (SL) were quantified. In *Thalictrum alpinum*, only the environment significantly influenced SD, with the variance component (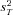) of the environment found to be much greater than that of genetics (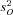) (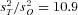). In *Kobresia humillis*, only genetics significantly influenced SD and SL, with the genetics variance component found to be greater than that of the environment (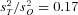, for SD). These results suggest that the extent to which genetics and the environment determine stomatal initiation and development is species-specific. This needs to be considered when studying genetic or environmental controls of stomatal initiation, as well as when SD and SL are used as proxies for ancient climate factors (e.g., CO_2_ concentration).

## Introduction

Stomata are the pores on the surface of leaves, flanked by guard cells, which regulate the gas exchange between internal plant tissues and the atmosphere, especially water vapor and CO_2_. Stomata are very important since they are directly responsible for the trade-off between water loss and carbon acquisition (Raven 2002). Gas exchange is controlled not only by the actual opening, but also the number and size of guard cells (stomatal density and length). Stomatal density (SD) and length (SL) are negatively correlated, a relationship that has seemingly existed for several hundreds of million years (Hetherington and Woodward 2003; Franks and Beerling 2009).

Generally, stomatal initiation is controlled by both environmental and genetic factors (Casson and Hetherington 2010). It is generally detected that SD and the concentration of atmospheric CO_2_ are inversely related, and thus the SD of fossil leaves has been used as a proxy indicator of paleoatmospheric CO_2_ levels (Woodward 1987; Royer 2001). On the other hand, SD, as a quantitative trait, is genetically determined (Gailing et al. 2008). Meantime, SL has been reported to correlate not only with genome size, but also with water conditions (Aasamaa et al. 2001; Beaulieu et al. 2008; Xu and Zhou 2008).

Some species have been reported as possessing generally high heritability (i.e., less sensitive to environmental change) in their stomatal characteristics (Sharma and Dunn 1969; Orlovic et al. 1998), while others have been reported as being more sensitive to environmental factors (Schoch et al. 1980). However, the relative importance of gene versus environment in determining SD or SL and its interspecific variation have not yet been estimated under a unified framework. Current knowledge regarding stomatal initiation comes from molecular biology, which depends heavily on a few model species, especially *Arabidopsis thaliana*. A lack of knowledge about the sensitivity to environmental factors of a trait within or between species limits the potential of that trait to be used in the reconstruction of paleoclimate (Royer et al. 2008, 2009).

In this study, we design in situ reciprocal transplant experiment along an elevation gradient to detect if the effect of genetic and environmental factors on stomatal density and length is species-specific.

## Materials and Methods

### Study site

The study was located at the Haibei Alpine Meadow Ecosystem Research Station (37°37′N, 101°12′E), Northwest Plateau Institute of Biology, Chinese Academy of Sciences. The station lies in the northeast of the Tibetan Plateau in a large valley surrounded by the Qilian Mountains. The climate there is a typical plateau continental climate, and is dominated by the southeast monsoon in summer and high pressure from Siberia in winter. The annual mean air temperature is −1.7°C and annual mean precipitation is 580 mm (Li et al. 2004).

### Experimental design

**Figure 1:**
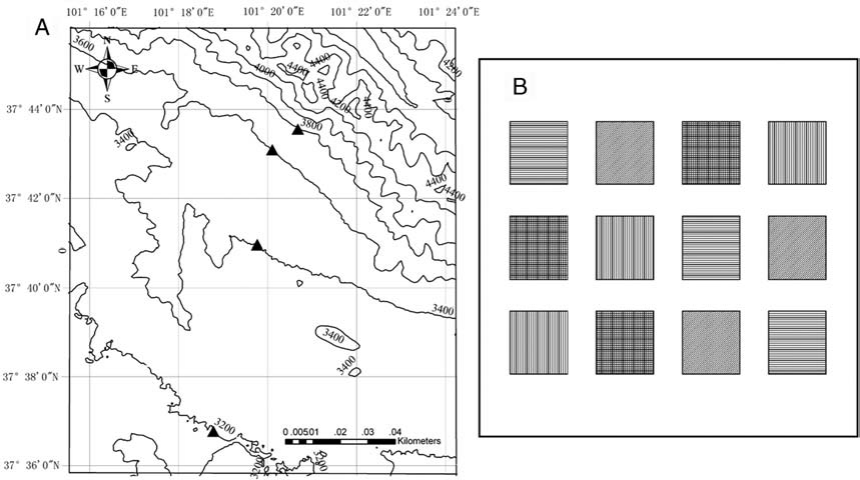
Location of the four altitudinal plots (A) and sketch-map of soil columns arranged at 3400 m (B). • represents the 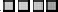 repsrepresent the soil columns originated from 3200 m, 3400 m, 3600 m and 3800 m.

Four elevations were chosen for an in situ reciprocal transplant experiment: 3200 m, 3400 m, 3600 m, and 3800 m (Fig. 1A). The difference in annual mean temperature between 3200 m and 3800 m was 2.4°C (2006–2008). The lapse rate during summer was estimated as −0.7°C per 100 m (Hirota et al. 2009). The communities at 3200 m and 3400 m were dominated by *Kobresia humilis* and *Potentilla fruticosa*, respectively. Meantime, *Carex moorcropt* dominated communities at both 3600 m and 3800 m.

At each elevation (original elevation), 12 soil columns measuring 1 × 1 × 0.3 m^3^ were excavated, and then transplanted to the four elevations (transplanting elevations). The procedure was executed with caution in order that the soil texture and vegetation in the columns remained intact. At each transplanting elevation, the 12 columns (three from the same elevation, nine from the other three elevations) were randomly assigned. When a column was transferred within the same elevation, its position was changed (Fig. 1B). The experiment began in May 2006.

### Stomatal density and stomatal length measurement

**Figure 2:**
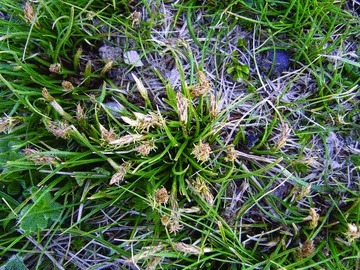
Plant of *K. humilis*. The photo is photographed by Xu Guangping.

*K. humilis* (Fig. 2) and *T. alpinum* were chosen because they were two among three common species present at all four elevations. But *K. humilis* did not appear at soil columns originated from 3400 m. Moreover, they were both hypostomatous (stomata on the abaxial surface). The two species are perennials with asexual propagation as the main way of annual regeneration (Deng et al. 2001). Therefore, it's easy to recognize the transplanted plants.

The plant material used for the SD and SL measurements was collected in August 2008. In each soil column, three plants were selected randomly and one fully mature leaf from each plant was selected and fixed immediately in FAA (the ratio of 70% ethanol, to ethanoic acid, to formalin was, by volume, 18:1:1). Finger polish imprints were taken from the whole of the abaxial surface of the basal leaflets for *T. alpinum* and mounted on a glass slide after the FAA solution had been removed. For *K. humilis*, the abaxial epidermis of the middle portion (Poole et al. 1996) of each leaf was scraped and mounted after the leaves had been softened in a 10% chromic acid solution. Five randomly selected fields of view (3–5 mm^2^) were selected to take images under a Motic microscope (Motic BA200, China). Using an image analysis system (Motic Images Advanced 3.2), SD was recorded at ×100 magnification. SL was taken as the length between the junctions of the guard cells at each end of the stoma. More than 30 stomata were randomly selected for SL measurement at ×100 magnification for *T. alpinum*, and at ×400 magnification for *K. humilis*.

### Statistical analysis

In order to compare the “background” variances between the two species in the spectrum of habitats they coexisted, a homogeneity test was conducted. Only the columns transferred within the same elevation were relevant to this test. The data were transferred by Box–Cox transformation.

Two-way analysis of variance (ANOVA) was used to analyze the effects of the environment (transplanting elevation), genetics (original elevation), and the interaction. Both the environment and genetic factors were set as random factors.

Variance decomposition in two-way ANOVA is given as 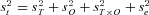, in which 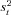 was the total variance, 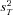 was the variance that originated from transplanting elevation, 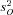 was the variance from the original elevation, 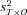 was the variance from the interaction between transplanting elevation and original elevation, and 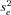 was the error variance. The variance of original elevation represented the effect of gene while the variance of transplanting elevation denoted the environmental effect (Strand and Weisner 2004).

In Minitab 15.0, the statistical package used, the variance components 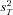 and 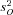 could be retrieved in the process of two-way ANOVA. The ratio 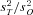 was calculated and submitted to *F*-test, as an extra estimate of the relative importance of environment to gene.

In two-way ANOVA, a soil column was taken as an experiment unit, i.e., all samples (three leaves per species) from one soil column were averaged into one data point. In homogeneity test, a plant was treated as an experimental unit; i.e., the five fields of view of a plant were averaged to form one estimator of the plant.

## Results

**Figure 3:**
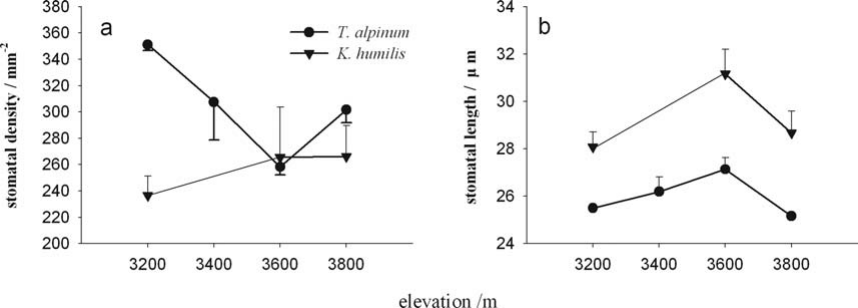
Stomatal density (a) and stomatal length (b) of *T. alpinum* (circles) and *K. humilis* (triangles) along elevation gradient.

Variations of SD and SL along the elevation gradient are shown in Figure 3. The tendency of SD along the elevation gradient is distinct between the two species (Fig. 3). SD of *T. alpinum* at 3200 m is the largest and the one at 3600 m is the smallest, while SD of *K. humilis* at 3800 m and 3200 m is the highest and the lowest (Fig. 3). On the contrary, SL of *T. alpinum* and *K. humilis* changes similarly (Fig. 3).

There is no significant difference between the two species in the “background” variance in both SD and SL (Table 1). The standard deviations and range (difference between the maximum and the minimum) of SD are quite close between the two species. The standard deviations and range of SL is higher in *K. humilis* than in *T. alpinum* (Table 1).

**Table 1:** Homogeneity test of *K. humilis* and *T. alpinum* along elevation gradient.

	SD				SL			
Species	Mean (±STD)	*N*	Range	*p*-value	Mean (±STD)	*N*	Range	*p*-value
*K. humilis*	256 (±65)	27	295		29.30 (±3.27)	27	13.22	
				0.437				0.272
*T. alpinum*	297 (±62)	21	208		26.11 (±1.68)	21	5.97	

Notes: Only the soil columns transplanted within the same elevation are used in the test. A single leaf is the basic unit of the test. SD is stomatal density, unit: number/mm^2^. SL is stomatal length, unit: μm. STD is standard deviation. *N* is the number of plants. Range is the maximum minus the minimum. *p*-value represents the probability of type I error based on Bartlett test with same variance as the null hypothesis.

ANOVA results from the reciprocal transplant experiment are given in Table 2. The results demonstrate that only the original elevation (genetic factors) had significant effects on the SD and SL of *K. humilis*, while only the transplant elevation (environmental factors) had significant effects on the SD of *T. alpinum*. The ratios of variance components (i.e.,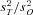) were calculated and tested by the *F*-test (Table 2). The two ratios for *T. alpinum* were less than 1 and the *p*-value for SD in *F*-test was 0.040, while those for *K. humilis* were greater than or equal to one and the *p*-value for SD was 0.091 (Table 2).

**Table 2:** Relative importance of original (genetic) and transplant (environmental) effects as represented by variance components in two-way ANOVA.

		*T. alpinum*				*K. humilis*			
		df	*F*	*p*-value	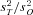	df	*F*	*p*-value	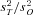
SD	T	3	5.75^*^	0.013		3	1.70	0.266	
	O	3	1.45	0.285	10.9^*^	2	6.51^*^	0.031	0.17^•^
	T × O	9	0.71	0.693		6	0.45	0.840	
SL	T	3	1.76	0.214		3	2.70	0.139	
	O	3	1.79	0.213	1.0	2	5.18^*^	0.049	0.52
	T × O	9	0.77	0.646		6	0.82	0.563	

Notes: The superscript “•” denotes statistical significance at the α= 0.10 level, and “^*^” denotes significance at the α= 0.05 level. SD is stomatal density, unit: number/mm^2^. SL is stomatal length, unit: μm. “T” represents environmental factors (transplant altitude) and “O” represents genetic factors (original altitude). “T × O” represents the interaction between the two factors, transplanted and original altitude. “df” stands for degrees of freedom. F is the ratio of treatment MS to error MS. “*p*-value” corresponds to *F* value. 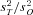 is the ratio of the two variance components, the transplant factor (environmental effect, numerator) and the original factor (genetic effect, denominator). The variance components were obtained as linear solutions of EMSs (expected mean squares) in ANOVA.

## Discussion

*T. alpinum* and *K. humilis* are species with wide distribution area and high genetic diversity. *T. alpinum* distributes from Asia-temperate to Asia-tropical and from Europe to Northern America (GRIN Taxonomy for Plants). Four varieties of *T. alpinum* have been recognized, two in China and two in America (Mooney and Johnson 1965). *K. humilis* is one of keystone species in meadow communities in Qinghai-Tibet Plateau. Zhao et al. (2006) investigated genetic diversity of *K. humilis* in eight populations in eastern Qinghai-Tibet Plateau using RAPD and discovered it had high diversity but most of the genetic variability (83.04%) resides among individuals within populations.

Genetic diversity or other biological factors constrain the spectrum of potential response. Before the effects of environment or genetics will be analyzed, the range of variation that is permitted by a species’ biology should be compared between the two species. For, if one species is permitted a higher degree of variation than another, the variances that could be induced by a same environmental stimulus would be different. Homogeneity test indicates that the two coexisting species along the altitudes possess similar degree of variation. Higher range and standard deviation of SL is found in *K. humilis* (Table 1). If this represented a higher degree of background variation that biology permitted, larger environmental effects on SL would be expected in *K. humilis* than in *T. alpinum*. This is, however, not the case. The environmental effects on SL is far from significant in *K. humilis*, while it is significant in *T. alpinum* (Table 2).

The relative importance of the environment (transplant elevation) and genetics (original elevation) in determining stomatal characteristics and variance between species has been estimated quantitatively under a unified framework. In terms of SD, an approximate 64-fold difference in ratio between the two species (Table 2) was found, while in terms of SL the difference was approximately twofold (Table 2). It seems that SD and SL in *T. alpinum* are more sensitive to environmental factors and *K. humilis* is influenced more by genetic factors when they originate from similar altitude backgrounds.

The responses of SD and SL to elevation gradient are diverse (Hovenden and Brodribb 2000; Hultine and Marshall 2000; Kouwenberg et al. 2007; Valladares et al. 2007; Holland and Richardson 2009). Likewise, contradictory results have been found regarding the responses of SD and SL to CO_2_ and water conditions and temperature (Aasamaa et al. 2001; Pearce et al. 2005; Zuo et al. 2005; Xu and Zhou 2008). For example, Ferris and Taylor (Ferris and Taylor 1994) reported that there were contrasting effects of elevated CO_2_ on SD among four grassland herbs. Consistent with these results, our study has quantified the relative importance of genetics and the environment. The controversy among these studies could be explained by the fact that the responses of stomatal characteristics to environmental changes were in fact species-specific. Therefore, this should be considered in cases where stomatal characteristics are being used as proxies for paleoclimatic factors as SD in some plants is not sensitive to environment change and the change along the altitude may not indicate fluctuation of CO_2_ concentration but background of gene especially in short-time terms (Royer 2001).

Advances have recently been made in our understanding of the regulation of stomatal development (Casson and Hetherington 2010). However, these studies have almost always been based on one model species, *Arabidopsis thaliana.* It seems that *A. thaliana* is a species with plasticity both in stomatal characteristics (Casson and Hetherington 2010) and other traits (Pigliucci 1998). A wider diversity of models for the genetic and environmental control of stoma should be considered.
